# Factor structure and psychometric evaluation of the Connor-Davidson resilience scale in a new employee population of China

**DOI:** 10.1186/s12888-017-1219-0

**Published:** 2017-02-02

**Authors:** Lei Wu, Yan Tan, Yan Liu

**Affiliations:** 1 Department of Epidemiology, Institute of Geriatrics, Chinese People’s Liberation Army General Hospital, Beijing, China; 2 Editorial Department, Academic Journal of Chinese People’s Liberation Army Medical School, Chinese People’s Liberation Army General Hospital, 28 Fuxing Road, Beijing, 100853 China; 3 Clinical Psychology, Beijing Huilongguan Hospital, Beijing, China

**Keywords:** New employee, Mental health. Resilience, Connor-Davidson resilience scale

## Abstract

**Background:**

In order to find a suitable instrument to evaluate psychological resilience in Chinese new employee population, we intended to propose a possible factorial structure of Connor-Davidson Resilience Scale (CD-RISC). Furthermore, we administered to explore its reliability and validity in the present population.

**Methods:**

Participants were derived from the male new employees who had started working in the last 2–3 months from 12 machinery factories across 8 different provinces of China. Chinese version of CD-RISC was used to assess the resilience of the study participants. Exploratory factor analysis (EFA) was conducted to assess the possible factor structure, and confirmatory factor analysis (CFA) was used to determine which factor structure was the most suitable among the present study sample.

**Results:**

The present 4-factor model (tolerance for stress, tenacity, and goal orientation; adaptability and acceptance of change; optimism and sense of security; and trust in one’s instinct) of CD-RISC showed good internal consistency, concurrent validity and consistent structure validity, and had presented better data fit than the original 5-factor and the Chinese 3-factor patterns. Each of the present 4-factor structure and the total score of CD-RISC were negatively and significantly associated with Global Severity Index T score and Personality Diagnostic Questionnaire-4+ score.

**Conclusions:**

The measure of resilience is useful in screening high-risk employees who are vulnerable to stress. Optimal and tailored interventions can be further applied to avoid potential adverse events in this population. Longitudinal research should be required to determine whether aging and long-term health events can change the nature of resilience.

**Electronic supplementary material:**

The online version of this article (doi:10.1186/s12888-017-1219-0) contains supplementary material, which is available to authorized users.

## Background

Initial stage of a job always subjects the junior workers to various types of stress, such as workloads, environmental changes, and interpersonal relationships. These sources of stress may lead to negative impacts on the young workers. A series of suicides and suicide attempts in a Chinese factory (resulting in 14 deaths) has suggested the importance of prevention of these adverse events [1]. Although some people experience negative mental health problems following exposure to adverse events, others may adapt well to the new surroundings [2]. Therefore, resilience has been widely concerned because its potential positive effects on reducing the impacts of stressful events, increasing the ability to maintain psychological and physiological functions, strengthening the capacity of recovering from the negative experiences, and actively coping with challenges [3–6].

Resilience is defined as the psychological characteristics that promote positive adaptation when facing stressful and adverse events [7]. Resilience is a multi-dimensional variable that changes in different populations with diverse living conditions, cultural background and socio-demographic characteristics. Early studies about psychological resilience have proposed different resiliency models [8, 9]. In 2003, a newly developed scale [10] has been proposed and then widely used in different countries [11–20]. When the scale was initially proposed by Connor and Davidson, it comprised 5 factors (the notion of personal competence, high standards, and tenacity; trust in one’s instincts, tolerance of negative effect, and strengthening effects of stress; the positive acceptance of change, and secure relationships; control; and spiritual influences) and 25 self-reported questions [10]. Some studies supported the original factorial framework [11, 12], whereas a large number of studies failed to support the original 5-factor analytic structure and proposed their factorial frameworks that fit in various countries [13–20]. For instance, Sexton et al. failed to support the scale by Connor and Davidson in participants with infertile experiences [17]. Lamond et al. also suggested that the scale was not supported in a community-dwelling older female population, and proposed a new 4-factor structure [14].

In China, Yu et al. translated the Connor-Davidson Resilience Scale (CD-RISC) into a Chinese version and instead yielded a 3-factor structure (Tenacity, Strength, and Optimism) in a Chinese community sample [19]. Recently, Xie et al. proposed another factorial framework in a Chinese military sample [20]. These findings indicated that the scale might need to be revised to meet the specific Chinese population. Therefore, we hypothesized that the present published factor structures might not be suitable for the new employee population, and we carried out a statistical analysis to determine whether the previous models [10, 19] of CD-RISC fit the data from a new employee population of China. In order to find a suitable instrument to evaluate psychological resilience in Chinese new employee population, we intended to propose a possible factorial structure of CD-RISC. Furthermore, we administered to explore its reliability and validity in the present population.

## Methods

### Study design

Details of the study design have been described in our previous study [21]. Briefly, a stratified clustering sampling method was performed in all machinery factories across 8 different provinces of China from September to December 2013. Employees were eligible to select following the standards: (1) physical healthy, without serious diseases; (2) had started working in the last 2–3 months; (3) were the first time to participate full-time job during the lifetime. Employees working in these factories need to perform a lot of manual labor. Of the selected 12 factories, a total of 3,997 participants completed the screening, and the response rate was 93.0%. All participants approved to complete the data collection and signed an informed consent. The proportion rate of the female workers was less than 5%; as a result, we only included the male workers. Finally, 3,960 eligible male subjects were included in the present analysis. The Independent Ethics Committee of the Chinese People’s Liberation Army General Hospital approved our project.

### Data collection

We organized these employees to complete a screening according to a standardized procedure. Each participant was asked to complete a self-reported questionnaire, including questions of personal characteristics (age, educational level, parental marital status, parental rearing patterns, only-child status, place of residence, and family income), CD-RISC questionnaire, Symptoms Checklist 90-Revised (SCL-90-R) questionnaire and Personality Diagnostic Questionnaire-4+ (PDQ-4+) questionnaire.

### Connor-Davidson resilience scale

The Chinese version of Connor-Davidson Resilience Scale (CD-RISC) questionnaire was used in our study [19]. The 25-item Guttman-scale (0 ‘not at all’, 1 ‘rarely true’, 2 ‘sometimes true’, 3 ‘often true’, 4 ‘extremely true’) was used to assess the resilience of the study sample with a total of 100 scores. A higher score indicates a higher level of resilience.

### SCL-90-R

Symptoms Checklist 90-Revised (SCL-90-R) was performed to examine the psychological symptoms status of the study sample. The SCL-90-R is a 90-item Guttman-scale (from 0 “not true at all” to 4 “extremely true”) self-reported questionnaire, which is used to screen the mental health symptoms [22]. The SCL-90-R has been translated into Chinese and proved to have good validity and reliability in different populations [23]. The global severity index (GSI) T score (mean score of all items of SCL-90-R) was reported in our analysis [24].

### PDQ-4+

Personality Diagnostic Questionnaire-4+ (PDQ-4+) questionnaire, a self-reported measurement of the Diagnostic and statistical manual of mental disorders (DSM), was used to screen the personality disorder (PD) traits of the study sample [25]. The total score of PDQ-4+ (each item ranges from 1 for “completely disagree”, to 5 for “completely agree”) was used in the present analysis. The PDQ-4+ has been widely used in Chinese population samples with good alpha coefficients [26].

### Statistical analysis

We used the Epidata (3.1) software to enter (double-entered) and check the data. SPSS software (Inc., Chicago, IL, USA), version 19.0 was used for the statistical analysis. Descriptive statistics were used to describe the characteristics of the study sample. All P values of <0.05 were defined statistically significant.

We randomly split the study sample (*n =* 3,960) into two random halves using a computerized statistical software program (SPSS software). First, Kaiser-Meier-Olkin test and Bartlett’s test of sphericity were conducted to explore whether the EFA was suitable to use in Sample 1 (*n =* 1,980). Parallel analysis (PA) was used to determine the number of extracted factors. The exploratory factor analysis (EFA) was then conducted to assess the possible factor structure using oblique rotation of Sample 1. Second, confirmatory factor analysis (CFA) was conducted in Sample 2 (*n =* 1,980) to examine the previous two factor structures (5-factor of original model and 3-factor of Chinese model) and the factor structure obtained from the EFA in Sample 1. SPSS AMOS software, version 17.0 was applied to test the goodness of fit between the observed data and the hypothesized models. The following fit indices were assessed: *χ* ^2^ */df* (the ratio of the chi-square to the degree of freedom), RMSEA (the root-mean-square error of approximation), GFI (the goodness-of-fit index), CFI (the comparative fit index), and TLI (the Tucker-Lewis index). The *χ* ^2^ */df* and RMSEA were less than 5 and 0.08 respectively; and the values of GFI, CFI and TLI of greater than 0.90 were viewed as acceptability criteria [27]. Third, Pearson correlations were calculated among each factor of CD-RISC, and between CD-RISC and theoretically related assessments (PDQ 4+ score and GSI T score). Furthermore, a multivariable linear regression model was used to identify the associations between score of each factor or total CD-RISC and the PDQ-4+ or GSI T score by calculating the adjusted β and the corresponding 95% confidence interval (CI).

## Results

### Description of the population

The characteristics of the study sample are presented in Table 1. A total of 3,960 male participants were included in our study. The mean (SD) age of the study participants was 18.7 (1.5) years, ranged from 16 to 24 years. About two-thirds of the participants had a lower educational level. Most participants were from double-parent family, lived in rural areas, and had a higher family income. About 50% of the participants were the only-child of the family, and 63.9% of the participants’ parental rearing pattern was democratic style. The means (SDs) of the CD-RISC score, SCL-90-R (GSI T score), and PDQ-4+ score were 63.4 (13.1), 50.0 (10.0), and 16.9 (10.9), respectively. The present study was a validation study based on cross-sectional data. The two random halves showed non-significant differences in all characteristics.

**Table 1:** Characteristics of the study participants

	Total (*n =* 3960)
Age (years), Mean (SD)	18.7 (1.5)
Educational level, n (%)	
High school and below	3085 (77.9)
College and above	875 (22.1)
Marital status of their parents, n (%)	
Married	3291 (83.1)
Divorced/Separated	669 (16.9)
Parental rearing pattern, n (%)	
Democratic	2532 (63.9)
Authoritative	720 (18.2)
Indulgent	560 (14.1)
Neglected	148 (3.7)
Single-child status, n (%)	
Singleton	1859 (46.9)
Non-singleton	2101 (53.1)
Place of residence before entering the factory, n (%)	
City	756 (19.1)
Rural	3204 (80.9)
Family income (Yuan/month), n (%)	
< 2000	1194 (30.2)
≥ 2000	2766 (69.8)
CD-RISC score, Mean (SD)	63.4 (13.1)
SCL-90-R (Global severity index T score), Mean (SD)	50.0 (10.0)
PDQ-4+ score, Mean (SD)	16.9 (10.9)

*SD* standard deviation, *CD-RISC* Connor-Davidson Resilience Scale, *SCL-90-R* Symptoms Checklist 90-Revised, *PDQ-4+* Personality Diagnostic Questionnaire-4+

### EFA of the CD-RISC

EFA was conducted to assess the possible factorial structure using oblique rotation in Sample 1 (*n =* 1,980). The coefficient of Kaiser-Meier-Olkin test was 0.961, and the Bartlett’s test of sphericity was statistical significant (*P <* 0.001). The scree plot was shown in Additional file 1: Figure S1. Score of each item, item-total correlations and the rotated factor pattern of the CD-RISC are shown in Table 2. The PA result indicated that the first 4 eigenvalues were greater than 1 and the factor loadings of all items were greater than 0.4. All items were positively related to the total score of CD-RISC, and the correlations ranged from 0.21 (item 20) to 0.72 (item 16). The percentages of variance explained by the 4 factors were 34.28% for factor 1, 7.13% for factor 2, 4.59% for factor 3, and 4.12% for factor 4. Factor 1 included items on tolerance for stress, tenacity, and goal orientation; factor 2 included items involving adaptability and acceptance of change; factor 3 reflected optimism and sense of security; and factor 4 corresponded to trust in one’s instinct.

**Table 2:** Score of each item, item-total correlation and oblique rotated factor pattern of the CD-RISC (*n =* 1,980)

Item	Score (mean ± SD)	Item-total correlation	Factor loadings			
			1	2	3	4
16	2.87 ± 0.87	0.72	0.77			
24	3.14 ± 0.84	0.65	0.73			
23	2.67 ± 1.01	0.65	0.73			
14	2.53 ± 0.94	0.67	0.72			
15	2.36 ± 1.00	0.69	0.71			
21	2.48 ± 0.98	0.65	0.71			
10	2.95 ± 0.84	0.62	0.68			
11	2.58 ± 0.84	0.63	0.67			
12	2.37 ± 0.98	0.59	0.65			
17	2.86 ± 0.97	0.64	0.64			
4	2.47 ± 0.87	0.62	0.61			
22	2.20 ± 1.11	0.54	0.57			
19	2.65 ± 0.94	0.61	0.56			
13	2.36 ± 1.02	0.60	0.55			
25	2.90 ± 1.01	0.49	0.41			
8	3.07 ± 0.89	0.66		0.72		
9	3.06 ± 0.94	0.42		0.68		
7	2.80 ± 0.90	0.68		0.67		
5	3.03 ± 0.88	0.66		0.66		
1	2.90 ± 0.89	0.62		0.62		
3	1.09 ± 1.02	0.27			0.71	
2	2.36 ± 1.23	0.44			0.68	
6	2.49 ± 0.94	0.53			0.60	
18	1.71 ± 0.93	0.24				0.78
20	1.52 ± 0.91	0.21				0.74
Eigenvalue			8.57	1.78	1.15	1.03
Variance explained (%)			34.28	7.13	4.59	4.12

*CD-RISC* Connor-Davidson Resilience Scale, *SD* standard deviation

### CFA for the CD-RISC with different models

**Fig. 1:**
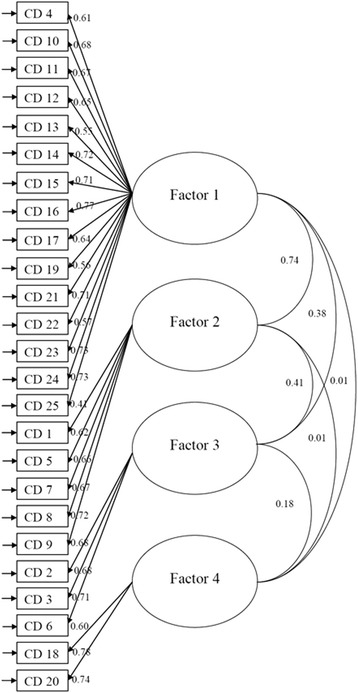
Graphical path diagram of the present 4-factor structure CD, Connor-Davidson

Details of the goodness-of-fit of the 3 models are shown in Table 3. CFA was performed to test the 5-factor of original model and the 3-factor of Chinese model of the CD-RISC in Sample 2 (*n =* 1,980). The *χ* ^2^ */df* and TLI of the original model were 14.58 and 0.88 respectively, failed to meet the acceptability criteria. The *χ* ^2^ */df,* CFI, and TLI of the Chinese model were 15.62, 0.89 and 0.88, respectively, did not meet the standard values. The above results revealed that these two models might not be supported by our study participants. For the present 4-factor model, the *χ* ^2^ */df* and RMSEA were 4.90 (<5.00) and 0.05 (<0.08) respectively, and the GFI, CFI and TLI were all greater than 0.90 in the present model, which indicated that the present model was supported by our study sample. Figure 1 presents the graphical path diagram of the present 4-factor structure of CD-RISC.

**Table 3:** Goodness-of-fit for the CD-RISC with different models

	*χ*2*/df*	RMSEA	GFI	CFI	TLI
Five-factor of original model	14.58	0.06	0.92	0.90	0.88
Three-factor of Chinese model	15.62	0.06	0.91	0.89	0.88
The present four-factor model	4.90	0.05	0.93	0.92	0.91

Table 4 shows the correlation coefficients of each factor and correlations of CD-RISC with the PDQ-4+ score, and the GSI score of the SCL-90-R in the present study participants. The total score and all 4 factors of the CD-RISC were negatively correlated with the GSIT score and the PDQ-4+ score (all P values <0.05). All 4 factors of CD-RISC were significantly related to the total score of CD-RISC.

**Table 4:** Correlation coefficients of each factor and correlations between CD-RISC and theoretically related assessments

	Factor 1	Factor 2	Factor 3	Factor 4	GSIT	PDQ-4+
CD-RISC	0.96^b^	0.84^b^	0.57^b^	0.16^b^	−0.35^b^	−0.28^b^
Factor 1		0.74^b^	0.38^b^	0.01	−0.39^b^	−0.32^b^
Factor 2			0.41^b^	0.01	−0.33^b^	−0.29^b^
Factor 3				0.18^b^	−0.06^b^	−0.03^a^
Factor 4					−0.15^b^	−0.17^b^

*PDQ-4+* Personality Diagnostic Questionnaire-4+ score, *GSIT* Global severity index T score, *CD-RISC* Connor-Davidson Resilience Scale

^a^Significant at the alpha 0.05 level

^b^Significant at the alpha 0.01 level

Additionally, the test-retest reliability was 0.676 for 1,533 participants who were randomly selected from the total participants across an interval of 3 months, and the Cronbach’s alpha for the full scale was 0.750.

### Association between resilience and mental symptoms or personality disorders

The multivariate linear regression analyses of the associations between the present 4-factor structure of CD-RISC and PDQ-4+ or GSI T score were shown in Table 5. After adjusting for age (continuous), educational level, marital status of their parents, parental rearing pattern, single-child status, place of residence before entering the factory, and family income, each factor and the total score of CD-RISC were significantly and negatively associated with GSI T score. The adjusted β (95% CI) of GSI T score was −0.27 (−0.29, −0.25) for the total CD-RISC score. Similarly, factor 1, 2, 4, and the total score of CD-RISC were significantly and negatively associated with PDQ-4+ score. The adjusted β (95% CI) of PDQ-4+ score was −0.23 (−0.26, −0.21) for the total CD-RISC score.

**Table 5:** Linear regression of the present 4-factor structure of CD-RISC

	GSI T score		PDQ-4+ score	
CD-RISC	β (95% CI)^a^	*P-*value	β (95% CI)^a^	*P-*value
Factor 1	−0.42 (−0.45, −0.39)	<0.001	−0.37 (−0.40, −0.33)	<0.001
Factor 2	−1.02 (−1.11, −0.92)	<0.001	−0.93 (−1.04, −0.83)	<0.001
Factor 3	−0.28 (−0.42, −0.14)	<0.001	−0.14 (−0.28, 0.14)	0.076
Factor 4	−0.98 (−1.19, −0.77)	<0.001	−1.22 (−1.44, −0.99)	<0.001
Total score	−0.27 (−0.29, −0.25)	<0.001	−0.23 (−0.26, −0.21)	<0.001

*CD-RISC* Connor-Davidson Resilience Scale, *PDQ-4+* Personality Diagnostic Questionnaire-4+, *GSI*, global severity index

^a^Each adjusted for age (continuous), educational level, marital status of their parents, parental rearing pattern, single-child status, place of residence before entering the factory, and family income

## Discussion

The present study used almost 4 thousand participants to assess the possible factorial structure and the reliability and validity of CD-RISC in a new employee population of China. We also collected the baseline demographic characteristics of the study participants, which made the possibility of the comparisons with other studies. The present 4-factor model showed good internal consistency, concurrent validity and consistent structure validity, and was proved superior to the original 5-factor and the Chinese 3-factor patterns in the present population. Furthermore, each of the present 4-factor structure and the total score of CD-RISC were negatively and significantly associated with the GSI T score and the PDQ-4+ score.

In agreement with several studies in this topic, our study did not support the original 5-factor model [13–20]. We proposed a new 4-factor structure, which has not been previously reported. Prior evidence has revealed that the resilience factors were widely varied in different populations [11–20]. This could be attributed to the fact that different countries have various cultural conventions and ethnic backgrounds. For example, as the least religious people in the world, Chinese people might have a different understanding of the item “sometimes fate or god can help”. Additionally, some researchers have pointed out the lack of robustness of the original factorial framework [28, 29]. Hubbard et al. reported that the solely usage of the Kaiser-Gutt-mann criteria to decide how many factors to retain might cause over-factoring [29]. Besides, our sample also failed to support the Chinese 3-factor model [19]. Different from our sample, the study by Yu et al. was administered to residents who engaged in different occupations. This indicates that the structure may require some modifications to adapt to specific populations.

We found that a 4-factor structure of the CD-RISC showed the best goodness of fit in the present study sample compared with the original 5-factor and the Chinese 3-factor patterns. Factor 1 occupies the majority of the explained variance of the present 4-factor structure. It extracts all items from the original 5-factor (factor 1 and 4), or includes a large number of items from the Chinese 3-factor (factor 1). This factor reflects that employees with higher psychological resilience integrate behaviors of tolerance for stress, tenacity, and goal orientation when facing adversity and frustration. Employees with resilient qualities can effectively address stressors and difficulties when doing manual labor in factory.

Factor 2 extracts all items from the Chinese version factor 2, or reflects quite a mixture of the original 5-factor structure. Similar to the process of disruption-reintegration [8], this factor reflects the adaptability and acceptance of environmental change in the resilient people. Factor 2 is a fundamental factor, because it not only contains the meaning of striving against difficult situations, but also reflects the basic meaning of resilience (bouncing back or positively recovering from the negative events) and positive psychology [30]. A Chinese worker with resilience can positively return to normal level of function when dealing with challenges.

Factor 3 mostly extracts the items from the Chinese version of factor 3, which reflects optimism and sense of security. As an important constitution of resilience, it presents the ability to confidently overcome the difficulties. Tindle et al. found that optimists had a lower hazard of coronary heart disease compared with pessimists, which revealed that interventions targeted to change attitudes might cause decreased health risks [31]. As a relatively young population, those resilient employees tend to face the uncontrollable events with positive mental attitude.

Compared with prior studies, the major difference of our study is the presence of factor 4. Factor 4 refers to the ability of trust in one’s instinct, which extracts a small number of items from the original factor 2, or the Chinese version of factor 1. This indicates the potential role of instinct among the employees in dealing with challenges. In agreement to our finding, Sprinks reported that staff on new course told to use gut instinct to avoid work stress [32]. This may infer the specific role of “trust in one’s instinct” among the employee population. It should be noted that the meaning of these items selected by Western researchers might be perceived different meanings to the present Chinese employees with relatively lower educational level. Additional studies should consider the populations with different cultural and educational backgrounds when applying the CD-RISC.

In the present study, we also demonstrated that each of the present 4-factor structure and the total score of CD-RISC were negatively and significantly associated with the GSI T score and the PDQ-4+ score. This finding not only proves the concurrent validity of the CD-RISC, but also reveals that the psychological resilience can be viewed as a protective factor against mental symptoms and personality disorder traits. In consistent with the study by Askeland et al., which reported that adolescents gained higher scores of resilience suffered from less mental health symptoms [33], the present findings indicate that proper interventions on the psychological resilience are likely to cause positive effects in the new employee population.

The present study contains several limitations. First, we used a cross-sectional study design to estimate the factorial structure of CD-RISC among the newly enrolled workers. This only limited by how an individual felt at the time of the measurement. Longitudinal comparisons of changes of CD-RISC should be studied in the future. Second, because the job in the factory involved a lot of manual labor, we only included the male workers with relatively lower educational level and income. Because of differences in culture, educational level and professional experience, the present results are likely difficult to generalize to other populations. However, the present 4-factor structure seemed suitable to the present population and could be used to screen employees with lower level of psychological resilience. Further studies are still required to examine the possible factor models of CD-RISC in populations of different age, gender, educational level and occupation. Third, a self-reported questionnaire was used to measure the constructs, and thus measurement bias cannot be avoided. For example, individuals in a negative emotional state may underestimate their psychological resilience and vice versa. Future work will benefit from integrating self-report with other methods to objectively measure psychological resilience.

## Conclusion

In summary, the present study proposed a 4-factor structure of CD-RISC and supported a suitable instrument to evaluate psychological resilience in Chinese new employee population. The measure of resilience is useful in screening high risk employees who are vulnerable to stress. Optimal and tailored interventions can be further applied to avoid potential adverse events in this population. Longitudinal research should be required to determine whether aging and long-term health events can change the nature of resilience.

## Additional file

Additional file 1: Figure S1. Scree plot of the present 4-factor structure. (DOCX 57 kb)

## References

[1] Cheng Q, Chen F, Yip PS (2011). The Foxconn suicides and their media prominence: is the weather effect applicable in China?. BMC Public Health.

[2] Tugade MM, Fredrickson BL (2004). Resilient individuals use positive emotions to bounce back from negative emotional experiences. J Pers Soc Psychol.

[3] Yi-Frazier JP, Smith RE, Vitaliano PP (2010). A person-focused analysis of resilience resources and coping in patients with diabetes. Stress Health.

[4] Seligman MEP, Csikszentmihalyi M (2000). Positive psychology. Am Psychologist.

[5] Werner E, Smith R (1992). Overcoming odds: high risk children from birth to adulthood.

[6] Rutter M (1985). Resilience in the face of adversity: protective factors and resilience to psychiatric disorders. Br J Psychiatry.

[7] Wagnild GM (2003). Resilience and successful aging: comparison among low and high income older adults. J Gerontol Nurs.

[8] Richardson GE (2002). The metatheory of resilience and resiliency. J Clin Psychol.

[9] Richardson GE, Neiger B, Jensen S (1990). The resiliency model. Health Educ.

[10] Connor KM, Davidson JR (2003). Development of a new resilience scale: the Connor-Davidson resilience scale (CD-RISC). Depress Anxiety.

[11] Yu XN, Lau JT, Mak WW (2011). Factor structure and psychometric properties of the Connor-Davidson resilience scale among Chinese adolescents. Compr Psychiatry.

[12] Jung YE, Min JA, Shin AY (2012). Positiveness research team of Korea. The Korean version of the Connor-Davidson resilience scale: an extended validation. Stress Health.

[13] Manzano-García G, Ayala Calvo JC (2013). Psychometric properties of Connor-Davidson resilience scale in a Spanish sample of entrepreneurs. Psicothema.

[14] Lamond AJ, Depp CA, Allison M (2008). Measurement and predictors of resilience among community-dwelling older women. J Psychiatr Res.

[15] Green KT, Hayward LC, Williams AM (2014). Mid-Atlantic mental illness research, education and clinical center workgroup. Examining the factor structure of the Connor-Davidson resilience scale (CD-RISC) in a post-9/11 U.S. Military veteran sample. Assessment.

[16] Mealer M, Schmiege SJ, Meek P (2016). The Connor-Davidson Resilience Scale in critical care nurses: a psychometric analysis. J Nurs Meas.

[17] Sexton MB, Byrd MR, von Kluge S (2010). Measuring resilience in women experiencing infertility using the CD-RISC: examining infertility-related stress, general distress, and coping styles. J Psychiatr Res.

[18] Jørgensen IE, Seedat S (2008). Factor structure of the Connor-Davidson resilience scale in South African adolescents. Int J Adolesc Med Health.

[19] Yu X, Zhang J (2007). Factor analysis and psychometric evaluation of the Connor-Davidson resilience scale (CD-RISC) in Chinese people. Soc Behav Pers.

[20] Xie Y, Peng L, Zuo X (2016). The psychometric evaluation of the Connor-Davidson resilience scale using a Chinese military sample. PLoS One.

[21] Tan Y, Liu Y, Wu L (2016). Screening results and correlates of personality disorder traits in a new employee population of China. Neuropsychiatr Dis Treat.

[22] Derogatis LR (1994). Symptom checklist-90-R: Administrative scoring and procedures manual.

[23] Tang QP, Tang ZH, Yuan AH (1999). The use and reanalysis of SCL −90 in China. Chinese J Clin Psych.

[24] Delara M, Woodgate RL (2015). Psychological distress and its correlates among university students: a cross-sectional study. J Pediatr Adolesc Gynecol.

[25] Hyler SE (1994). PDQ-4+ personality questionnaire.

[26] Yang J, McCrae RR, Costa PT (2000). The cross-cultural generalizability of axis-II constructs: an evaluation of two personality disorder assessment instruments in the Peoples republic of china. J Personal Disord.

[27] Hooper D, Coughlan J, Mullen M (2008). Structural equation modelling: guidelines for determining model fit. Dublin Inst Techno.

[28] Khoshouei MS (2009). Psychometric evaluation of the Connor-Davidson resilience scale (CD-RISC) using Iranian students. Int J Test.

[29] Hubbard R, Allen SJ (1987). An empirical comparison of alternative methods for principal component extraction. J Bus Res.

[30] Friedman SE, Baum N (2016). The role of positive psychology in the modern medical practice. J Med Pract Manage.

[31] Tindle HA, Chang YF, Kuller LH (2009). Optimism, cynical hostility, and incident coronary heart disease and mortality in the Women’s Health Initiative. Circulation.

[32] Sprinks J (2012). Staff on new course told to use ‘gut instinct’ to avoid work stress. Nurs Stand.

[33] Askeland KG, Hysing M, Aarø LE (2015). Mental health problems and resilience in international adoptees: Results from a population-based study of Norwegian adolescents aged 16–19 years. J Adolesc.

