# Supplementary figures and images for: Factor structure and psychometric evaluation of the Connor-Davidson resilience scale in a new employee population of China
Source: BMC Psychiatry. 2017 Feb 2;17:49. doi: 10.1186/s12888-017-1219-0 (PMC5290619; doi:10.1186/s12888-017-1219-0)

**
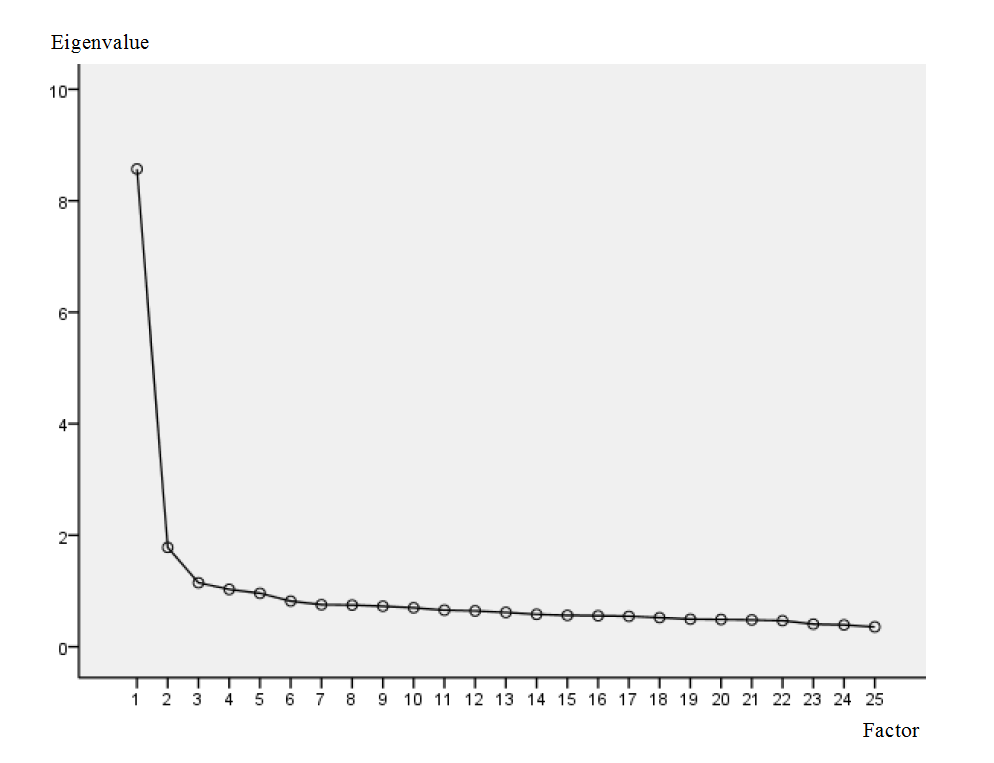
**

**Supplementary Figure 1** Scree plot of the present 4-factor structure

Supplement: Additional file 1: Figure S1. — Scree plot of the present 4-factor structure. (DOCX 57 kb) [file 12888_2017_1219_MOESM1_ESM.docx]
